# Uncovering and Correcting Shortcut Learning in Machine Learning Models for Skin Cancer Diagnosis

**DOI:** 10.3390/diagnostics12010040

**Published:** 2021-12-24

**Authors:** Meike Nauta, Ricky Walsh, Adam Dubowski, Christin Seifert

**Affiliations:** 1 Faculty of EEEMCS, University of Twente, 7500 AE Enschede, The Netherlands; 2 Institute for Artificial Intelligence in Medicine, University of Duisburg-Essen, 45131 Essen, Germany; 3 Cancer Research Center Cologne Essen (CCCE), 45147 Essen, Germany

**Keywords:** deep learning, explainable AI, skin cancer diagnosis, inpainting, shortcut learning, model bias, confounding

## Abstract

Machine learning models have been successfully applied for analysis of skin images. However, due to the black box nature of such deep learning models, it is difficult to understand their underlying reasoning. This prevents a human from validating whether the model is right for the right reasons. Spurious correlations and other biases in data can cause a model to base its predictions on such artefacts rather than on the true relevant information. These learned shortcuts can in turn cause incorrect performance estimates and can result in unexpected outcomes when the model is applied in clinical practice. This study presents a method to detect and quantify this shortcut learning in trained classifiers for skin cancer diagnosis, since it is known that dermoscopy images can contain artefacts. Specifically, we train a standard VGG16-based skin cancer classifier on the public ISIC dataset, for which colour calibration charts (elliptical, coloured patches) occur only in benign images and not in malignant ones. Our methodology artificially inserts those patches and uses inpainting to automatically remove patches from images to assess the changes in predictions. We find that our standard classifier partly bases its predictions of benign images on the presence of such a coloured patch. More importantly, by artificially inserting coloured patches into malignant images, we show that shortcut learning results in a significant increase in misdiagnoses, making the classifier unreliable when used in clinical practice. With our results, we, therefore, want to increase awareness of the risks of using black box machine learning models trained on potentially biased datasets. Finally, we present a model-agnostic method to neutralise shortcut learning by removing the bias in the training dataset by exchanging coloured patches with benign skin tissue using image inpainting and re-training the classifier on this de-biased dataset.

## 1. Introduction

In recent years, deep learning methods have been applied to a variety of problems with promising results. From speech recognition [1] and autonomous driving [2] to image-based cancer detection [3], deep neural networks (DNNs) have often outperformed traditional machine learning methods [4,5,6]. In 2019, Esteva and Topol outlined the transformative potential of AI for skin cancer diagnosis [7]. Furthermore, Google recently announced an AI tool for dermatological diagnosis, which proves that this subject is quite topical [8,9].

**Figure 1:**
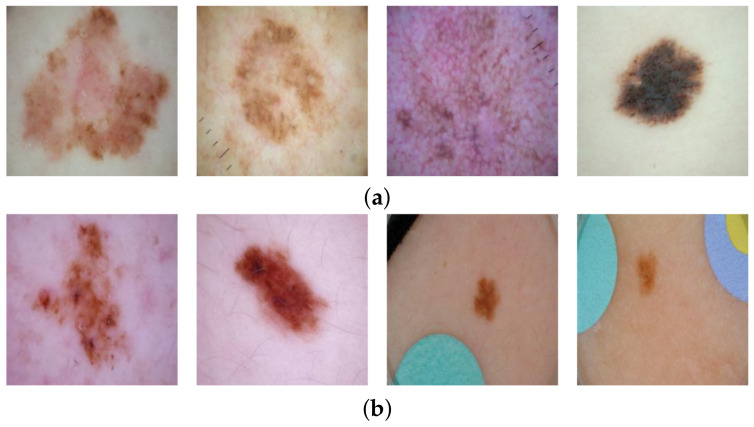
Examples from the ISIC dataset. Half of the benign lesion images include a coloured patch in the image which could cause a classifier to rely on these patches. (**a**) Malignant, no images with coloured patches in the dataset; (**b**) Benign, approximately half of the images show coloured patches.

Higher predictive accuracy comes at a cost, however, as these models are often black boxes [10]. The lack of model interpretability means that domain experts cannot check the underlying reasoning of a predictive model. In particular, it is often difficult to determine whether a model is a so-called “Clever Hans” predictor [11]: producing seemingly correct results during training which rely on spurious correlations in the training data and not does not rely on any relevant rule. Those learned “shortcuts” [12] might work well on standard benchmark datasets but usually fail to transfer to real world data. Shortcut learning can, therefore, cause incorrect performance estimates. As concluded by Geirhos et al. [12], “we must not confuse performance on a dataset with the acquisition of an underlying ability”. It is known that various artefacts can be present in dermoscopy images [13], increasing the risk of shortcut learning. For example, surgical skin markings in images can adversely impact skin cancer classification performance [14]. Moreover, the widely used dataset for skin cancer classification from the International Skin Imaging Collaboration (ISIC) [15] contains artefacts: Half of the images of benign lesions contain elliptical, coloured patches (colour calibration charts [13]), whereas the malignant lesion images contain none (cf. Figure 1). Hence, a deep learning model could learn to associate a coloured patch with a benign lesion and exploit this spurious correlation to achieve an artificially high prediction accuracy. Thus, in the first instance, the reported accuracy of such a shortcut model is higher than what would be expected when patches are no longer available. Furthermore, if a malignant lesion appears “in the wild” alongside a coloured patch (for example, when the diagnosis is not known a priori), then the model may base its decision on the patch, resulting in an incorrect diagnosis. It is, therefore, essential that biases are unearthed to avoid unexpected outcomes after deployment.

In this work, we examine how often a standard classification model for diagnosing skin cancer learns shortcuts. We present a methodology to measure and quantify shortcut learning in an already trained model and present a method to remove this confounding bias. More specifically, we will study the following research questions:

*RQ1:*  To what extent is a standard skin cancer classifier taking shortcuts by using the coloured patches in the ISIC dataset for prediction?
*RQ2:*  To what extent can the undesired usage of artefacts be removed by replacing the patches in the ISIC dataset with automatic inpainting and retraining the classifier?

In order to address these research questions, we apply automatic inpainting techniques. Image inpainting, also known as image completion, is the task of reconstructing missing regions in an image by estimating suitable pixel values. By masking the coloured patches in the ISIC data and letting an inpainting model automatically fill these masks, coloured patches are replaced by skin-coloured pixels. We then compare the difference in predictions of a classifier on the original benign images and on the images where those patches were replaced with benign skin to analyse to what extent the classifier relies on coloured patches. Similarly, we insert coloured patches into images with malignant skin lesions and subsequently compare the predictions of the classifier on the original and altered images to assess the risk that a malignant image with a coloured patch will be misclassified as benign. These analyses constitute our answers to *RQ1*. We emphasise that we do not change the training procedure of the original classifier but only pass original or modified images through a trained classifier to evaluate to what extent the classifier relies on shortcuts. This makes our approach model agnostic.

We further investigate to what extent we can remedy the undesired behaviour of the classifier by automatically inpainting all patches in the training data with benign skin and re-training the classifier on this de-biased dataset (*RQ2*). Since our approach removes artefacts from the data, it is classifier agnostic and can, therefore, be applied to other model architectures as well.

In summary, the contributions of this paper are as follows:

- We propose a model-agnostic method to quantify the extent to which a classifier relies on artefacts present in the training data (“shortcuts”) by using inpainting methods.
- We apply and validate the method on the ISIC skin cancer dataset.
- By removing the artefacts with in-distribution inpainted images, we show that we can train an unbiased classifier.

The rest of the paper is organised as follows. The subsequent section details the background and related work on interpreting deep learning models and uncovering biases. Section 3 describes the dataset and introduces our methodology to evaluate and quantify shortcut learning. Results are presented in Section 4, and they are then discussed in Section 5. In Section 6, we draw conclusions from the main findings.

## 2. Related Work

Common deep learning models are nowadays black boxes by nature. Assessing and understanding the reasoning of models is the focus of research in explainable artificial intelligence (X-AI) [16]. Explainability is especially important when algorithms are applied for medical diagnosis in order to establish trust among the medical community [17]. Notably, in 2014, Zeiler and Fergus [18] published a seminal paper explaining the predictions of convolutional neural networks (CNNs). One of their methods involves systematically occluding sections of the input images with grey boxes and assessing the change in predicted probabilities. Bazzani et al. [19] also used occluded images with grey boxes for unsupervised object localisation. Issues may arise with this method, however, as regions of grey pixels have not been observed by CNN during training and such occluded images may, therefore, be out-of-distribution, raising questions about the reliability of the results [20].

The approach by Fong and Vedaldi [21] was to introduce blur and noise to parts of the image and again assess the change in predictions. The authors noted that this method could introduce artefacts, thus producing nonsensical results if the CNN has not observed examples of blur during training. Unexpected results from “fooling” neural networks has been studied by, e.g., Nguyen et al. [22], who found that CNNs sometimes believe with high confidence that images of pure static are recognisable objects. Chang et al. [23] and Burns et al. [20] addressed this issue. Instead of grey boxes or image perturbations, both of these studies used inpainting to replace sections of an image, i.e., pixel patterns that fit with the rest of the image, in order to understand what the most important features were for a specific model prediction. We also apply inpainting, but rather than assessing important features for individual images (local explanations [16]), we seek to identify across the full training dataset whether a CNN is using spurious correlations in order to uncover shortcut learning on a global level.

Rieger et al. [24] studied confounders in the ISIC dataset, namely the presence of coloured patches in benign skin images. Where Bissoto et al. [25] already found that skin lesion classifiers rely on these patches by occluding images with black patches, Rieger et al. [24] proposed a method to solve this shortcut learning by lowering the importance of the patch regions during training. By defining a preferred region of interest and penalising the classifier for focusing on the patch regions, it optimises the model towards paying attention to the lesion and not to artefacts. Moreover, follow-up work by Bissoto et al. [26] aimed to de-bias the machine learning model. In contrast, we propose an alternative method of training a shortcut-free classifier by adapting the dataset instead of the classifier or its training procedure. This means that our methodology is model-agnostic and is not tied to any specific machine learning model or architecture. Moreover, we can assess a classification model after it has already been trained by presenting a method to measure and quantify the amount of shortcut learning by a trained model. This means that existing models that might already be operational in clinical practice can be tested for shortcut learning.

## 3. Materials and Methods

### 3.1. Data Set and Classifier

We retrieved skin lesion images from the ISIC Archive Dataset [15] using the code provided by Rieger et al. [24]. In total, 2506 images labelled as cancerous (malignant) and 19,298 non-cancer (benign) images were retrieved. Coloured patches were present in 8973 (46%) of the benign images but none were present in malignant images. Example images are shown in Figure 1. We randomly split the data set into training (80%) and test set (20%) and used the same splits for training the inpainting model and the classification model.

In order to analyse shortcut learning in classifiers for diagnosing skin cancer, we first train a neural network to distinguish between benign and malignant lesions. We emphasize that our goal is not to train a new, state-of-the-art classifier but instead to analyse the impact of shortcut learning in a widely used classification model. We followed the methodology by Rieger et al. [24], which allows us to compare with the results of that study. We used VGG16 [27] as the classifier, a standard neural network architecture which is also previously used for skin cancer diagnosis [28,29]. VGG16 consists of a feature extractor with 13 convolutional and pooling layers and 3 fully connected layers for the classification. The output layer has two nodes: one for predicting the probability that an image contains a malignant lesion and one for predicting benign. For details regarding this architecture, we refer to the original publication [27] or to a discussion on neural networks for melanoma detection [28]. We applied a standard training strategy by using a pre-trained VGG16 from Pytorch [30], freezing the weights for the feature extraction layers and only adapting the final classification layers to our data set (the source code for our methodology is available at https://github.com/adubowski/shortcuts-skin-cancer, accessed on 17 November 2021). We chose the same hyperparameters as Rieger et al. [24] by relying on the associated source code (https://github.com/laura-rieger/deep-explanation-penalization, accessed on 17 November 2021): training with SGD for 10 epochs with a learning rate of 0.00001 and momentum of 0.9.

The raw images of the ISIC dataset have differing resolutions. We resize all images to 224×224, which is the standard image resolution for VGG16 [27,29,31], and this allows us to use pre-trained weights for the VGG16 model. (Note that Rieger et al. [24] used the non-standard resolution of 299×299. For completeness, we also trained a VGG16 model on the non-standard 299×299 size but we found no significant differences in classification accuracy compared to the standard 224×224).

### 3.2. Removal of Coloured Patches for Benign Cases

For automatic inpainting, we used the Generative Multi-column Convolutional Neural Network (GMCNN) model by Wang et al. [32]. This inpainting model requires a masked image as input, after which it is trained with reconstruction loss to predict suitable pixel values to fill in the hole in the image. We set the hyperparameters of the inpainting model as the authors proposed it in their source code (https://github.com/shepnerd/inpainting_gmcnn, accessed on 17 November 2021). The model, which had been pretrained on celebrity faces with rectangular masks, was finetuned on our dataset for 25 epochs, since the authors of the inpainting model advise finetuning for 20–40 epochs on a relatively small dataset size similar to ours. Better inpainting results may have been possible after training longer than 25 epochs, but we were limited in computing resources.

Inpainting models require binary masks indicating the regions the model should fill. In order to train the inpainting model on our dataset, we created random elliptical masks close to the boundaries of the images. Randomness was introduced to the locations, sizes and shapes of these masks, but they conformed roughly to the shapes of the observed coloured patches. Only images without patches were used for training to ensure that the model learns to inpaint skin only.

**Validity Check.** In order to evaluate inpainting quality and validate that the presence of inpainted pixels does not result in unexpected output of the classifier, we applied two validity checks. As the basis for these checks, we take the images *without* coloured patches in the test set and use the GMCNN model to automatically inpaint random elliptical sections in the image. The first check calculates the structural similarity index measure (SSIM) [33] between each original image and its corresponding randomly inpainted image. An SSIM of exactly 1 would indicate perfect inpainting. We use the implementation in scikit-image [34] with parameters set to match the implementation of Wang et al. [33]. Secondly, we analyse the behaviour of the classifier on these inpainted images by assessing the changes in output probability. This validity check evaluates whether inpainting results in a distribution shift for the classifier. If the predictions for each original image and inpainted image are similar, we can conclude that the inpainting model works reasonably well and that the inpainted regions are indeed “uninformative” [20] replacements.

**Patch Removal.** In order to mask the coloured patches present in some images in the dataset, we need to know the exact location of these patches. As described by Rieger et al. [24], the coloured patches can be segmented using the SLIC image-segmentation algorithm [35] and splitting the segments based on the difference in RGB and HSV values to an average skin tone. Since Rieger et al. have publicly shared masks from their study, these have been re-used for the current study. However, we found that these masks did not always completely cover the coloured patches. Since this might lead the inpainting model to take the remaining pixels of the coloured patch and fill the hole with the original colour of the patch, thereby “repairing” the patch instead of removing it, we additionally dilated the masks before inpainting. For this, we used an elliptical structuring element of size 9×9 pixels such that the coloured patch was covered by the mask while not drastically increasing the mask size. Dilation was implemented using OpenCV [36].

**Figure 2:**
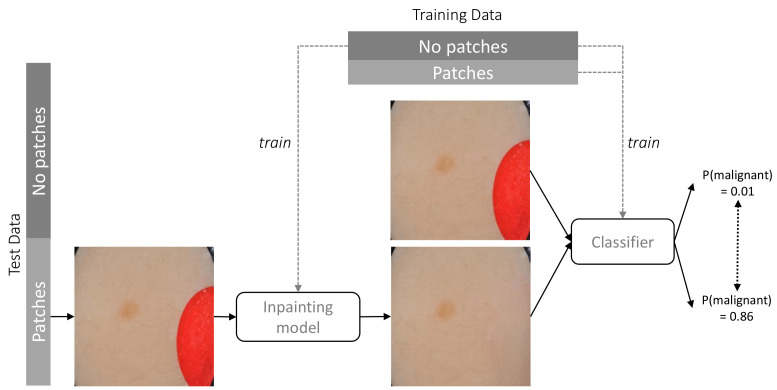
Methodology for assessing the extent to which the classifier relies on coloured patches for predicting a benign case. The classifier is trained on the full training set, the inpainting model is trained with training images without patches. The classifier is tested on both the unaltered test set and after replacing patches with inpainted skin.

In order to assess the extent to which the classifier relies on coloured patches for predicting a benign case, we apply the trained inpainting model to the subset of test set images containing coloured patches. The trained inpainting model replaces these patches with artificially generated skin, after which these altered images are forwarded through the classifier. By comparing the difference between the prediction for the original image with patch and the prediction for the inpainted image without patch, we can measure how much the classifier relies on the coloured patch for each image. By aggregating over all test images with a patch, we can quantify the extent to which the classifier bases its decisions on those artefacts. Figure 2 shows an overview of this approach. Similarly to the inpainting validity check above, we calculate the Structural Similarity Index Measure (SSIM) in order to evaluate the difference between the altered images and their original counterparts.

### 3.3. Inserting Coloured Patches to Malignant Cases

**Figure 3:**
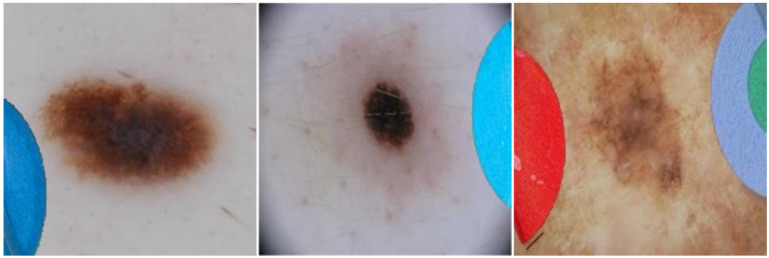
Three examples of images of malignant lesions with coloured patches inserted to evaluate whether the presence of a patch changes the prediction of the classifier.

In order to assess the risk of classifying a malignant image as benign due to the presence of coloured patches, we add such patches to malignant images. We used the same segmentation approach as described in the previous section (Section 3.2) to extract patches from benign images and inserted them into images of malignant lesions, as shown in Figure 3. Again, class probabilities were obtained from the classifier and were compared for the original malignant images vs. the altered versions including patches. We observed that in some cases the inserted coloured patches overlapped part of the lesions, which may affect classifier results. We manually removed these images to obtain 361 (73%) of the 492 malignant images in the test set for evaluation. For completeness, we compared the results when using all malignant images in the set and observed no significant differences.

Additionally, we again calculate SSIM between the original image and the modified image. This indicates how much the insertion of patches changes the images, which can be compared with the changes in predictions by the classifier.

### 3.4. Quantifying the Classifier’s Dependency on Coloured Patches

By passing both the original test images as well as the altered test images (either with patches inserted or removed) through the classifier, two sets of predictions can be compared. We emphasise that we do not *train* the classifier at this step but forward the images through the already trained model to collect its predictions. In order to quantify how much the classifier relies upon the absence or presence of the coloured patches (the “shortcut”), we calculate the Mean Absolute Deviation (MAD). This metric indicates how much, on average, modifying the image (i.e., inserting or removing a patch) affects the predicted class probability by the classifier.

When passing an input image x with label y∈{benign,malignant} through classifier *C* (in our case a VGG16 model), *C* outputs probability P(malignant|x) and probability P(benign|x), which sum up to 1. We denote with $x_{patch}$ an image containing a coloured patch, and $x_{\setminus patch}$ is an image without a coloured patch in the original dataset. We denote with $x_{+patch}^{\prime }$ an altered version of $x_{\setminus patch}$ where a coloured patch was initially absent but was artificially inserted. Similarly, $x_{-patch}^{\prime }$ is an image where a coloured patch was initially present but was removed with inpainting. In order to quantify how much classifier *C* relies on the coloured patches to correctly classify a benign lesion, we calculate the following:

(1) $${\mathrm{MAD}}_{{\mathrm{benign}}^{\setminus patch}}=\frac{1}{\left|A\right|}\sum_{x_{patch}\in A}\left|P\left(\mathrm{benign}|x_{-patch}^{\prime }\right)-P\left(\mathrm{benign}|x_{patch}\right)\right|,$$

where A denotes the set of benign images with coloured patches (i.e., $x_{patch},y=\mathrm{benign}$) in the test set. In order to quantify how much the model relies on the coloured patches when classifying malignant images with coloured patches, we calculate the following:

(2) $${\mathrm{MAD}}_{{\mathrm{malignant}}^{+patch}}=\frac{1}{\left|B\right|}\sum_{x_{\setminus patch}\in B}\left|P\left(\mathrm{malignant}|x_{+patch}^{\prime }\right)-P\left(\mathrm{malignant}|x_{\setminus patch}\right)\right|,$$

where B denotes the set of malignant images without coloured patches (i.e., $x_{\setminus patch},y=\mathrm{malignant}$) in the test set.

The MAD score does not contain information on the *direction* of change. We wish to assess for what fraction of benign images the predicted probability of being benign *decreases* and for what fraction of malignant images the predicted probability of being malignant decreases (denoted as %P↓), respectively. Therefore, we additionally take the non-absolute deviation into account and calculate the fraction of images ∈A with respect to B for which the deviation was negative and report the mean negative deviation (MND) for this fraction. Lastly, we calculate the fraction of images ∈A with respect to B for which their predictions crossed the decision boundary and, hence, where the modification would have resulted in a different classification. This score indicates the risk that a malignant image with a coloured patch would be classified as benign.

### 3.5. Inpainting to Create a Dataset without Artefacts

In addition to quantifying shortcut learning, it is desirable to fix shortcut learning and thereby remove the impact of unwanted confounders. Hence, for our second research question *RQ2*, we address this issue by replacing the confounders in the training dataset (in our case the coloured patches in the ISIC data) with inpainting and retrain the classifier on this de-biased dataset. Specifically, we first train the classification model as described in Section 3.1 on the unaltered training data (“Vanilla Classifier”). We then finetune the inpainting model on this training set, excluding images with patches. In this manner, the inpainting model is trained to generate skin-coloured segments without coloured patches. This approach is similar to the evaluation methodology presented before (cf. Section 3.2), but instead of applying it to the test set only, we augment the *training dataset* by replacing the coloured patches with their inpainted counterparts. In this manner, a classifier cannot learn a relationship between coloured patches and benign lesions and might generalise better in the real world. We train a classifier with the same architecture as before on this altered training data (“Retrained Classifier”). We apply the same three experiments as described in Section 3.2 and Section 3.3: a validity check for the inpainting model, quantifying shortcut learning of the classifier when coloured patches are removed and quantifying shortcut learning when coloured patches are inserted.

Additionally, we assess the overall classification performance of the retrained classifier since ideally the retraining does not result in a decrease in performance. In addition to comparing performance, we want to ensure that there are no major changes in predictions between the retrained classifier and original vanilla classifier. Thus, to assess the fidelity in predictions between the two classifiers, we visualise the predicted probabilities P(malignant|x) for individual examples as a scatterplot and calculate Pearson’s correlation coefficient between the two sets of predicted probabilities.

## 4. Results

In this section, we evaluate to what extent the classifier used the coloured patches in the dataset as shortcuts by the classifier by adding or removing those patches in the dermoscopy images of the ISIC dataset. In Section 4.1, we first validate that inpainting does not result in a distribution shift by randomly inpainting segments of images. Section 4.2 and Section 4.3 evaluate shortcut learning by either removing coloured patches in benign images with inpainting (as presented in Section 3.2) or inserting coloured patches in malignant images (as presented in Section 3.3). Lastly, Section 4.4 compares the performance of the original classifier with a classifier retrained on the de-biased dataset, following the methodology presented in Section 3.5.

Table 1 already summarises the main results, where each row will be discussed in more detail in the corresponding subsections. The reported sensitivity (true positive rate) and specificity (true negative rate) are given by a probability threshold of P(malignant|x)=0.4. This threshold was selected based on observing the distribution of predicted probabilities for either class and to ensure a higher sensitivity so as to capture more cancerous lesions.

### 4.1. Evaluation of the Inpainting Model

**Figure 4:**
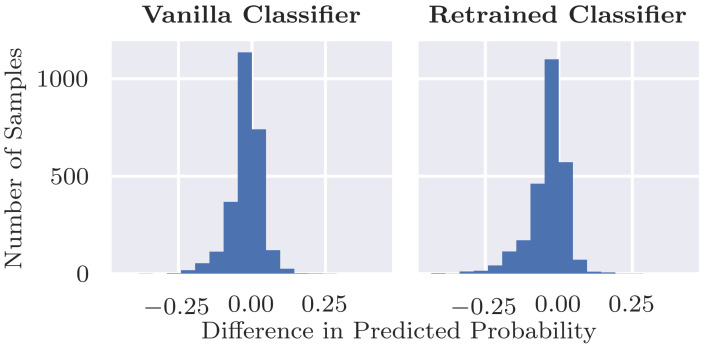
Differences for P(malignant|x) before and after inpainting a random segment in test images without a coloured patch.

The validity check experiment described in Section 3.2 evaluates the inpainting model and involves inpainting random sections in test set images without coloured patches. Table 2 shows that the Structural Similarity Index Measure is almost equal to 1, indicating that the randomly inpainted image is very similar to its original counterpart. Secondly, we assessed the changes in output probability from the classifier. Figure 4 shows that, for both classifiers, the differences in probabilities before and after inpainting centre are around zero, indicating that in general the inpainting has little effect on the class predictions. There is a slight skew to the left, however, suggesting that inpainting is more likely to result in a decrease in class probability (i.e., closer to benign) than an increase, and this is investigated below. Table 1 further shows that there is little change in overall performance after random inpainting, albeit with a slight decrease in sensitivity from 0.902 to 0.870 and a slight increase in specificity. The results are broadly similar when using the classifier retrained after replacing coloured patches with inpainting.

**Figure 5:**
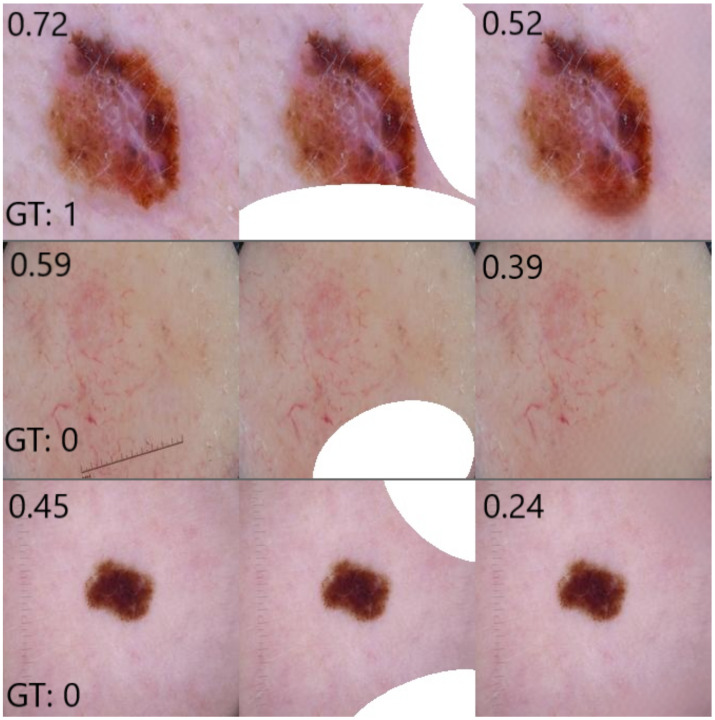
Examples of outliers with large probability drops after inpainting random sections. Shown are the original lesions, random masks and inpainted results, with the predicted probabilities P(malignant|x) and the ground truth (GT) labels (0 = benign; 1 = malignant).

Only for a small fraction of the samples, inpainting a random region results in a drop in probability P(malignant|x) of 0.2 or greater, as shown in Figure 4. We can observe three main groups of outliers with a large drop in probability after inpainting, which are shown in Figure 5. By manual inspection, we observed that many outliers are similar to the top example shown where the lesion is partially covered by inpainting. Inpainting, thus, changes the shape or characteristics of the lesion; thus, a change in class probability is not unreasonable. There are a small number of cases where the inpainting of ruler markings results in a drop in probability as in the second example shown. This is an interesting example indicating the possible presence of another bias in the dataset as ruler markings occur more frequently with malignant lesions, as pointed out by Rieger et al. in their Supplementary Materials [24]. There are also several cases such as the third example where there is no clear reason for the drop in probability. These are fewer in number but may be indicative of an issue in the robustness of the classifier. However, based on the observed results, we can conclude that inpainting does not always result in an *increase* in predicted probability but that the probability stays similar for most cases and also slightly decreases for a few. Hence, we can conclude that the inpainting model works reasonably well and that our approach is a valid method to assess classification results after inpainting coloured patches.

### 4.2. Removal of Coloured Patches for Benign Cases

**Figure 6:**
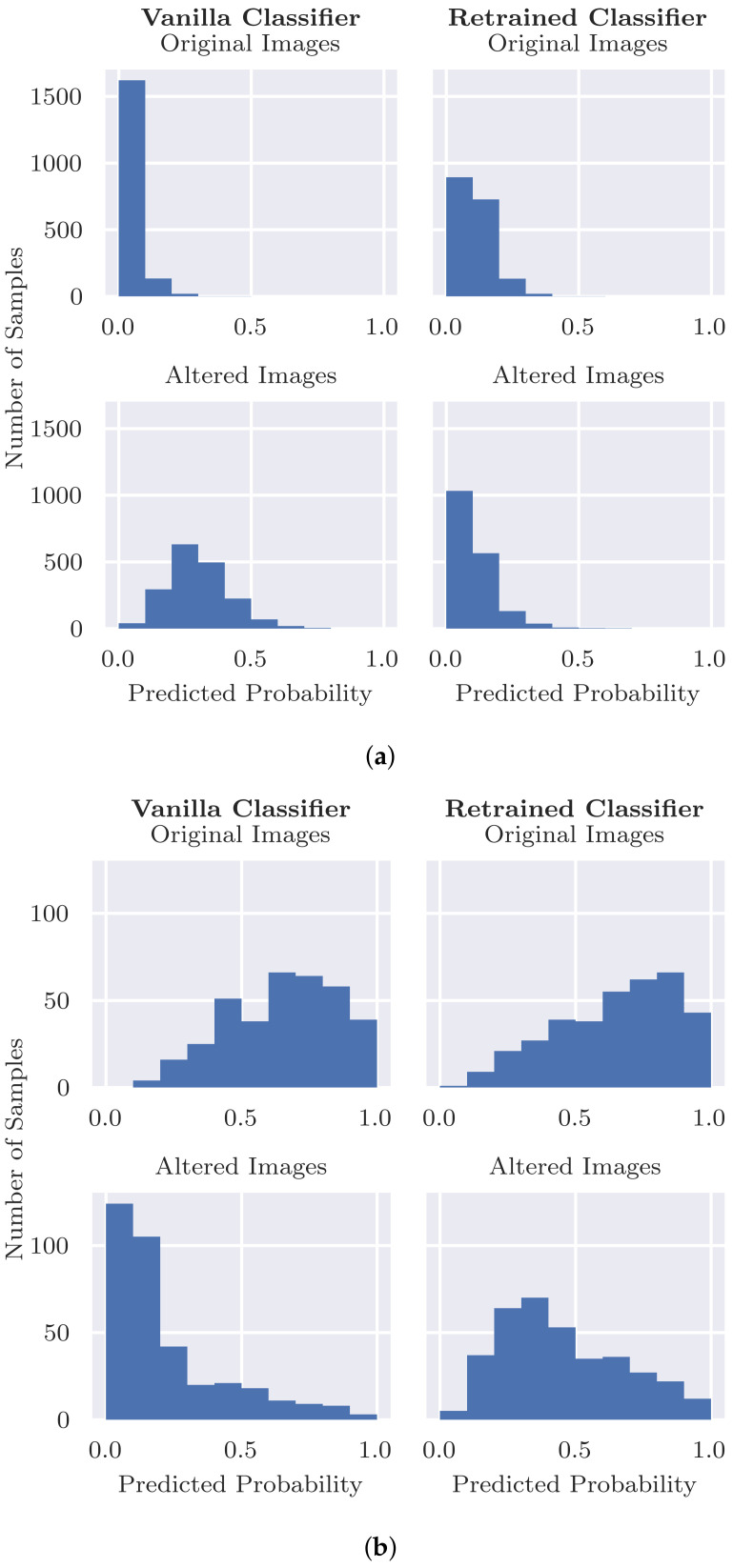
Predictions P(malignant|x) by the classifiers before (Original) and after (Altered) removal resp. insertion. (**a**) Inpainting the coloured patches in benign images. Only images with a patch are included; (**b**) inserting coloured patches into images of malignant lesions.

Figure 6a shows that the vanilla classifier originally predicts probabilities very close to zero (i.e., being benign) when coloured patches are present. The probability distribution shifts towards the malignant class when the patches are removed using inpainting (bottom row Figure 6a). Although the original images and their inpainted versions are still relatively similar, as shown by the high SSIM in Table 2, the response by the classifier on such a modest change is striking. In Table 3, the top row presents more detailed results. The predicted probability of 99.7% of the benign images with patches moves towards the malignant class after inpainting. Of those images, the average difference in predicted probability after inpainting is 0.268, which is lower than the decision boundary. This means that the decision boundary P(malignant|x)=0.4 is crossed for 21.1% of the images, meaning that those images would be misclassified as being malignant. Table 1 shows that removing the patches from the images causes specificity to decrease from 0.999 to 0.788. These results indicate that the classifier is using the coloured patches to some extent, as the probability distribution moves towards the malignant class, but it is apparently still able to use other information in the images to make a correct decision for most of the images.

When the patches have been removed from the training set through inpainting and the classifier has been retrained, we observe that the reliance on coloured patches significantly decreased. Figure 6a shows that the majority of probabilities remain below the decision boundary for the original images (with patches) and the altered (inpainted) images when using the Retrained Classifier. Furthermore, Table 3 shows that for the vanilla classifier, predicted probabilities decreased by an average of 0.26 after inpainting the patches. In contrast, the differences in predicted probabilities for the retrained classifier is almost zero, meaning that this classifier does not rely on using the presence of coloured patches to predict benign. Specifically, initially the retrained classifier misclassifies only 0.1% of the benign images. After inpainting the patches in the test set, in addition, 0.6% of the benign images were incorrectly classified as being malignant, which is substantially lower than for the vanilla classifier. Table 1 also reflects this, where specificity only decreases from 0.999 to 0.993 after removing patches in the test set.

### 4.3. Inserting Coloured Patches to Malignant Cases

Figure 6b shows that adding a coloured patch to a malignant image decreases the probability of being identified as malignant for both the vanilla and retrained classifiers. However, the effect was much more significant in the case of the vanilla classifier. It is especially striking to observe that the vanilla classifier is very confident, and the malignant images with coloured patches inserted are benign. In contrast, the retrained classifier has its confidence more centered around the decision boundary. Table 3 shows that the probability decreases by an average of 0.4 with an inserted patch for the vanilla classifier but an average decrease of roughly 0.2 for the retrained classifier. Sixty-nine point five percent (69.5%) of the malignant images that were initially correctly classified are, after inserting patches, incorrectly classified as benign. This behaviour also results in a large drop in sensitivity for the vanilla classifier from 0.886 to 0.191, as reported in Table 1. For the retrained classifier, the number of misclassifications caused by inserting patches reduces substantially but is, at 33.5%, still not close to zero. The retrained classifier also observes a drop in sensitivity, although not as extreme, from 0.839 to 0.512.

**Figure 7:**
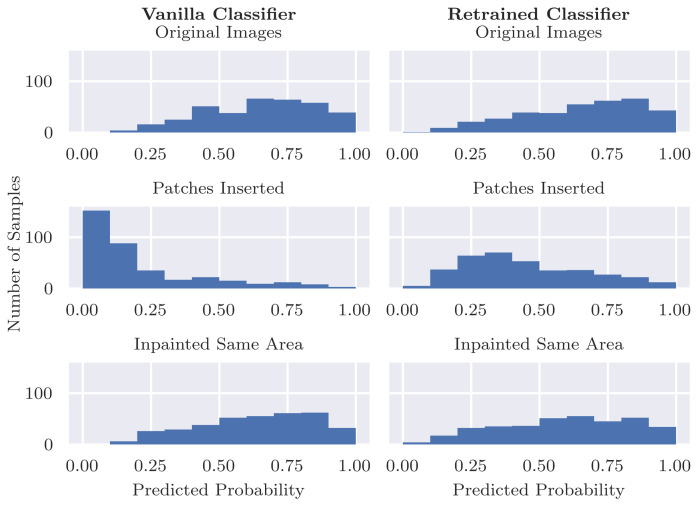
Predicted P(malignant|x) for original malignant images, after inserting patches, and after inpainting the same area that the patch covers. The probability distribution for the inpainted images is closer to that of the original images rather than the images with inserted patches.

**Figure 8:**
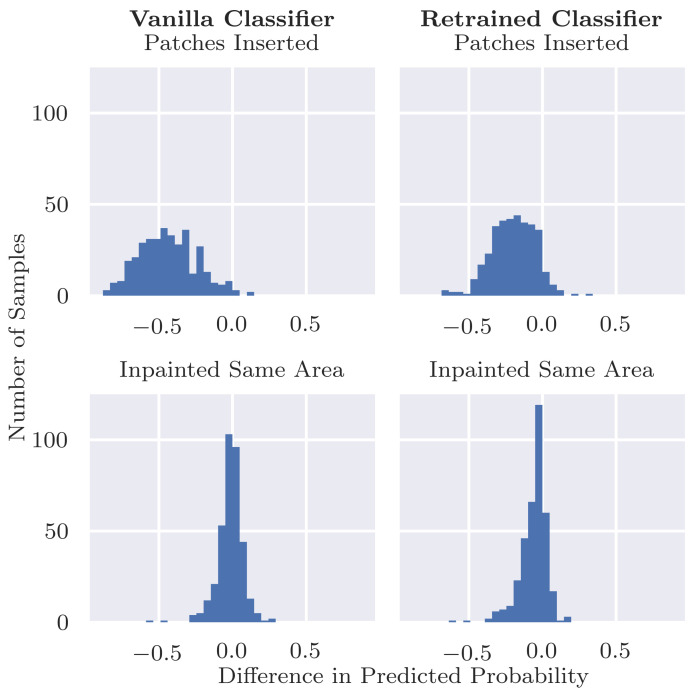
Differences in P(malignant|x) from the original malignant images after inserting patches (top row) or inpainting the same area as the patches (bottom row). The changes in probabilities for the inpainted images are much closer to zero than for the images with inserted patches, indicating that the patches themselves are causing the drop in predicted probability rather than the area that they are covering.

Since the retrained classifier was not trained on images with coloured patches, it is an unexpected result that also the retrained classifier shows a significant drop in predicted probability after inserting patches into malignant images. One plausible hypothesis is that the patches cover important information in the image when they are inserted. Although we included a manual step that excluded inserted patches that clearly covered the lesion, we evaluate this hypothesis also in an automated manner. Specifically, we inpaint the region that was covered by the inserted patch and forward this image through the classifier. If the patches were indeed covering relevant pixels in the image, then we would expect to observe similar drops in probability when these regions are inpainted. However, Figure 7 and Figure 8 show that these large drops in probability do not occur for the inpainted examples. This indicates that the inserted patches themselves are responsible for the large drops in probability and not their location.

### 4.4. Classifier Performance after Retraining

The results from Section 4.2 and Section 4.3 have shown that a classifier trained on the inpainted dataset reduces its reliance on coloured patches. By de-biasing the classifier, we hypothesised that the classifier is able to better learn “the right reasons” for distinguishing between benign and malignant without sacrificing classification accuracy. The top two rows in Table 1 present sensitivity and specificity of both the vanilla classifier and the retrained classifier on the complete test set and a test set where we exclude images with coloured patches. The latter set is included to evaluate the vanilla and retrained classifier for images where the confounder (i.e., coloured patches) is not present. We can observe that overall performance does not differ much between the classifiers, confirming that the training dataset contains enough patterns to predict the right class without having to rely on coloured patches.

**Figure 9:**
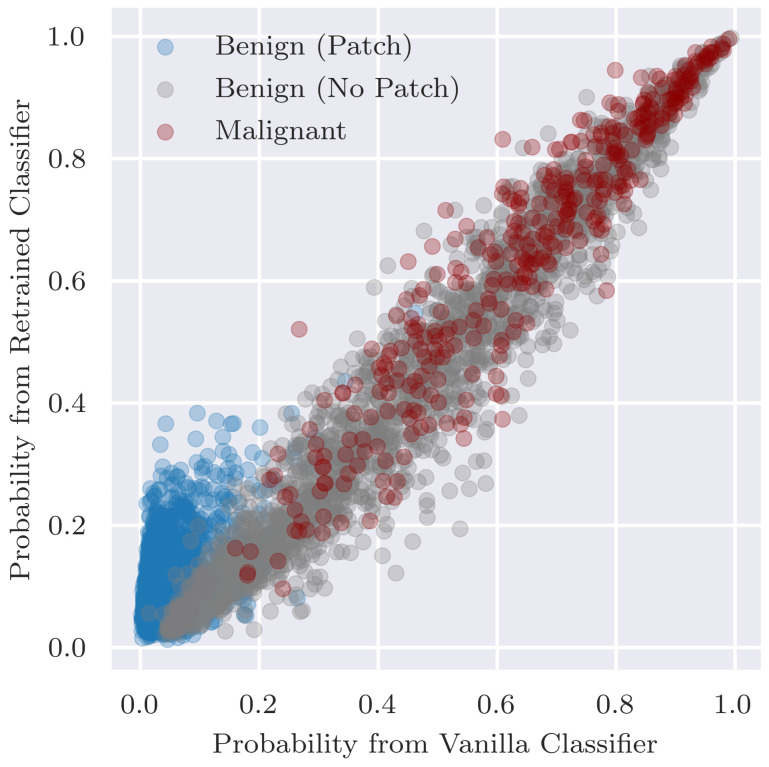
Predictions of P(malignant|x) from the vanilla classifier vs. classifier that has been retrained by replacing patches with inpainting.

Figure 9 shows how predictions for individual examples change when the classifier is retrained on an altered training set where the coloured patches are replaced with inpainting. Overall, there is good agreement and the Pearson’s correlation coefficient is r = 0.96. For the malignant images (r = 0.95) and benign images with no patches (r = 0.97), the predictions lie close to a diagonal line and agree well between both classifiers. For images with patches, however, there is less agreement (r = 0.52), and we can observe that the predicted probability tends to increase when we remove the patches from the training set images. This is to be expected given that the initial classifier was able to rely on the presence of coloured patches to confidently predict that the lesion was benign.

Much of our classifier implementation is based on the study of Rieger et al. [24], who report an AUC of 0.93 and F1 score of 0.56 on the entire unaltered ISIC test set (results were updated by the authors from the published version to the results in arXiv:1909.13584v4), compared to AUC = 0.89 and F1 = 0.49, respectively, for our study. They also record a modest increase in performance after implementing their method for fixing the bias during training, whereas no meaningful prediction difference between our vanilla classifier and retrained classifier was observed.

## 5. Discussion

*Quantifying the classifier’s reliance on artefacts (RQ1):* Our results show that our standard classifier is using patches as shortcuts when making predictions for skin cancer. We first evaluated how much the model relies on these patches to correctly predict benign lesions. Even when the patches are removed, the model is able to use other information in these images to correctly predict benign lesions in many cases, albeit with a drop in specificity as the model becomes less confident. Thus, in many cases we can say that the model is “right for the right reasons”. However, when we compare the specificity for images with patches vs. those with no patches in the original test set, we see a big difference both for the unaltered images and inpainted versions. Thus, it is possible that patches tend to occur more frequently in “easier” images, i.e., those that are more clearly benign. If the patches occurred instead in more difficult cases, then we might expect a heavier reliance on the patches to predict benign lesions.

The converse and arguably more serious case is examined by our second evaluation on inserting patches into malignant images. While patches occur only in benign images in this pre-labelled dataset, there is no guarantee that this will be the case in future clinical practice. Our results showed the potentially dangerous nature of the bias, where the presence of a coloured patch alongside a malignant lesion (Figure 3) resulted in a sizeable drop in sensitivity from 0.886 to 0.191. As shown in Table 3, this means that 69.5% of malignant lesions pictured next to a coloured patch may be misdiagnosed as benign because of the bias in training data and the shortcuts learned by the classifier. When such a shortcut model would be used in clinical practice, this can result in missed treatment opportunities and potentially fatal outcomes.

*Can inpainting be used to remedy the undesired usage of artefacts in the training set (RQ2):* Adjusting the training set by replacing the coloured patches with inpainted skin pixels certainly reduces the bias of the model. Particularly promising is the retrained classifier’s ability to correctly classify benign images with patches regardless of whether the patch has been inpainted or not. From the validity check that randomly inpaints images that have no patches, it does not seem that the model is associating the presence of inpainted pixels with the benign class. Although the predicted probability P(malignant|x) is more likely to decrease than increase with inpainting, the results were similar for the vanilla classifier for which larger decreases in probability tended to be caused by the inpainting covering part of the lesion or some other salient information.

However, the issue with misdiagnosed malignant lesions alongside coloured patches was not fully resolved by the retrained model. The sensitivity dropped from 0.839 to 0.512 when patches were inserted, which, although not as severe as for the original vanilla classifier, is still significant. A potential cause of this behaviour is that the patches are “out-of-distribution” with respect to the training data, resulting in unreliable predictions by the classifier. Further analysis could help to shed more light on the cause, e.g., using class activation maps or analysing the types of patches that result in the largest changes.

We note that we may just be replacing one confounder in the data, the coloured patches, with another artifact in the form of inpainted regions, and the model could detect inpainted patterns and learn to associate them with benign lesions. There is little evidence of this behaviour in the current study, as when we compared predictions of inpainted test images vs. the original unaltered images, no strong bias was observed from the retrained classifier. This might change, however, with a more sophisticated classification model. If the problem arose, then a possible solution would be to include randomly inpainted versions of images during training.

*Limitations and Practical Implications:* Inpainting proved to be a useful technique for understanding model predictions, and this work builds on the existing results in this area [20,23]. The main difference in the current study is to use predefined masks for inpainting which replace specific biases, rather than previous studies which inpaint different sections of the image to detect the most salient regions. The main benefit of our approach is that this allows the overall effect of specific biases to be assessed easily without having to examine many saliency maps for individual images. In particular, our quantitative metrics are useful for summarizing the extent of shortcut learning across a dataset. The main limitation with this, however, is that the bias in the data needs to be easily identifiable and able to be segmented from the images.

The method of reducing bias proposed in the current study by retraining on inpainted results is applied to the data only and is independent of classification models. Thus, it has the advantage that it is not restricted to specific neural network architectures or even other machine learning methods in contrast to many existing methods [24,37,38]. It does, however, have the extra burden of training an inpainting model for replacing the bias and this may not work for every problem and dataset.

## 6. Conclusions

We presented a methodology based on inpainting to analyse and quantify to what extent a skin cancer classifier is basing its decisions on wrong reasons (i.e., “shortcut learning [12]”). We found that inpainting is a viable method of assessing the overall impact of a bias in a training dataset for deep learning models. Specifically, we showed that a standard classifier trained on the ISIC dataset to predict skin cancer from images relies on shortcuts by making use of a bias in the dataset. Since coloured patches were only present in benign images, the classifier uses this shortcut to make its prediction. Such behaviour is undesired when the model is used in clinical practice, since we showed that inserting a coloured patch next to a malignant lesion can result in a benign prediction. Hence, shortcut learning can result in a serious risk of misdiagnoses. Replacing the source of bias in the training images by automatically inpainting those areas was found to decrease the reliance on shortcuts by the classifier. Although not perfect, inpainting models provide a sufficient level of detail to remove unwanted aspects in images such as the coloured patches present in the ISIC dataset.

With our results, we would like to raise awareness of the risks of using black boxes trained on datasets with potential biases. We hope that our research proves useful for researchers wishing to assess and measure the potential behaviour of their models, and we hope that our study provides a method for reducing the potential impact of biases. Potential further work might include extending the methodology to other biases or other datasets and experimenting with different inpainting models.

## Figures and Tables

**Table 1:** Performance of classifiers on specific subsets of the test set when trained on the original training set (“Vanilla”) or trained on the augmented training set where coloured patches are inpainted (“Retrained”). Note that Sensitivity or Specificity are indicated with “n.a” when the specific data subset contains samples of only one class (benign resp. malignant).

	Vanilla Classifier		Retrained Classifier	
Data Subsets	Sensitivity	Specificity	Sensitivity	Specificity
Full Test Set	**0.902**	0.750	0.868	**0.775**
Test Set excl. Images with Patches	**0.902**	0.537	0.868	**0.586**
Random Inpainted Test Set	**0.870**	0.559	0.793	**0.632**
Only Benign with Patches	n.a.	0.999	n.a.	0.999
Only Benign with Inpainted Patches	n.a.	0.788	n.a.	**0.993**
Only Malignant in Test Set	**0.886**	n.a.	0.839	n.a.
Only Malignant with Inserted Patches	0.191	n.a.	**0.512**	n.a.

**Table 2:** Structural Similarity Index Measure (SSIM) between the altered images and their original versions. A value closer to 1 indicates that the images are more similar.

Data Subsets	Median SSIM	Mean SSIM ± Std. Dev.
Random Inpainted Test Set	0.978	0.970 ± 0.024
Benign with Patches Inpainted	0.906	0.899 ± 0.057
Malignant with Patches Inserted	0.925	0.911 ± 0.060

**Table 3:** Quantification of learned shortcuts on the test set for the original classifier (“Vanilla”) and the retrained classifier trained on the training set where coloured patches are inpainted (“Retrained”). MAD: mean absolute deviation of predictions after removing resp. inserting patches. %P↓: fraction of images where probability of true class decreases. MND: mean negative deviation. %flip: fraction of images where the prediction after removal/insertion crossed the decision boundary. Lower is better for all metrics.

Data Subsets	Classifier	MAD	%P↓	MND	%flip
Benign with Patches Removed	Vanilla Classifier	0.268	99.7	0.268	21.1
	Retrained Classifier	**0.044**	**40.4**	**0.047**	**0.6**
Malignant with Patches Inserted	Vanilla Classifier	0.462	98.9	0.466	69.5
	Retrained Classifier	**0.206**	**93.4**	**0.216**	**33.5**

## Data Availability

Skin lesion images were retrieved via the API from the ISIC Archive Dataset [15].

## References

[1] Nassif A.B., Shahin I., Attili I., Azzeh M., Shaalan K. (2019). Speech Recognition Using Deep Neural Networks: A Systematic Review. IEEE Access.

[2] Grigorescu S., Trasnea B., Cocias T., Macesanu G. (2020). A survey of deep learning techniques for autonomous driving. J. Field Robot..

[3] Hu Z., Tang J., Wang Z., Zhang K., Zhang L., Sun Q. (2018). Deep learning for image-based cancer detection and diagnosis—A survey. Pattern Recognit..

[4] Mohammadi M., Al-Fuqaha A., Sorour S., Guizani M. (2018). Deep learning for IoT big data and streaming analytics: A survey. IEEE Commun. Surv. Tutor..

[5] Lenselink E.B., Ten Dijke N., Bongers B., Papadatos G., Van Vlijmen H.W., Kowalczyk W., IJzerman A.P., Van Westen G.J. (2017). Beyond the hype: Deep neural networks outperform established methods using a ChEMBL bioactivity benchmark set. J. Cheminform..

[6] Coccia M. (2020). Deep learning technology for improving cancer care in society: New directions in cancer imaging driven by artificial intelligence. Technol. Soc..

[7] Esteva A., Topol E. (2019). Can skin cancer diagnosis be transformed by AI?. Lancet.

[8] Bui P., Liu Y. (2021). Using AI to Help Find Answers to Common Skin Conditions (The Keyword|Google). https://blog.google/technology/health/ai-dermatology-preview-io-2021/.

[9] Jain A., Way D., Gupta V., Gao Y., de Oliveira Marinho G., Hartford J., Sayres R., Kanada K., Eng C., Nagpal K. (2021). Development and Assessment of an Artificial Intelligence–Based Tool for Skin Condition Diagnosis by Primary Care Physicians and Nurse Practitioners in Teledermatology Practices. JAMA Netw. Open.

[10] Barredo Arrieta A., Díaz-Rodríguez N., Del Ser J., Bennetot A., Tabik S., Barbado A., Garcia S., Gil-Lopez S., Molina D., Benjamins R. (2020). Explainable Artificial Intelligence (XAI): Concepts, taxonomies, opportunities and challenges toward responsible AI. Inf. Fusion.

[11] Lapuschkin S., Wäldchen S., Binder A., Montavon G., Samek W., Müller K.R. (2019). Unmasking Clever Hans predictors and assessing what machines really learn. Nat. Commun..

[12] Geirhos R., Jacobsen J.H., Michaelis C., Zemel R., Brendel W., Bethge M., Wichmann F.A. (2020). Shortcut learning in deep neural networks. Nat. Mach. Intell..

[13] Mishra N.K., Celebi M.E. (2016). An overview of melanoma detection in dermoscopy images using image processing and machine learning. arXiv.

[14] Winkler J.K., Fink C., Toberer F., Enk A., Deinlein T., Hofmann-Wellenhof R., Thomas L., Lallas A., Blum A., Stolz W. (2019). Association between surgical skin markings in dermoscopic images and diagnostic performance of a deep learning convolutional neural network for melanoma recognition. JAMA Dermatol..

[15] Codella N., Rotemberg V., Tschandl P., Celebi M.E., Dusza S., Gutman D., Helba B., Kalloo A., Liopyris K., Marchetti M. (2019). Skin Lesion Analysis Toward Melanoma Detection 2018: A Challenge Hosted by the International Skin Imaging Collaboration (ISIC). arXiv.

[16] Guidotti R., Monreale A., Ruggieri S., Turini F., Giannotti F., Pedreschi D. (2018). A survey of methods for explaining black box models. ACM Comput. Surv. (CSUR).

[17] Tjoa E., Guan C. (2021). A survey on explainable artificial intelligence (xai): Toward medical xai. IEEE Trans. Neural Netw. Learn. Syst..

[18] Zeiler M.D., Fergus R. Visualizing and Understanding Convolutional Networks. Proceedings of the European Conference on Computer Vision.

[19] Bazzani L., Bergamo A., Anguelov D., Torresani L. Self-taught object localization with deep networks. Proceedings of the 2016 IEEE Winter Conference on Applications of Computer Vision (WACV).

[20] Burns C., Thomason J., Tansey W. Interpreting black box models via hypothesis testing. Proceedings of the 2020 ACM-IMS on Foundations of Data Science Conference.

[21] Fong R.C., Vedaldi A. Interpretable explanations of black boxes by meaningful perturbation. Proceedings of the IEEE International Conference on Computer Vision.

[22] Nguyen A., Yosinski J., Clune J. Deep neural networks are easily fooled: High confidence predictions for unrecognizable images. Proceedings of the IEEE Conference on Computer Vision and Pattern Recognition.

[23] Chang C.H., Creager E., Goldenberg A., Duvenaud D. Explaining Image Classifiers by Counterfactual Generation. Proceedings of the International Conference on Learning Representations.

[24] Rieger L., Singh C., Murdoch W., Yu B. Interpretations are Useful: Penalizing Explanations to Align Neural Networks with Prior Knowledge. Proceedings of the International Conference on Machine Learning, PMLR.

[25] Bissoto A., Fornaciali M., Valle E., Avila S. (De) Constructing bias on skin lesion datasets. Proceedings of the IEEE/CVF Conference on Computer Vision and Pattern Recognition Workshops.

[26] Bissoto A., Valle E., Avila S. Debiasing Skin Lesion Datasets and Models? Not So Fast. Proceedings of the IEEE/CVF Conference on Computer Vision and Pattern Recognition (CVPR) Workshops.

[27] Simonyan K., Zisserman A. (2014). Very deep convolutional networks for large-scale image recognition. arXiv.

[28] Hosseinzadeh Kassani S., Hosseinzadeh Kassani P. (2019). A comparative study of deep learning architectures on melanoma detection. Tissue Cell.

[29] Nahata H., Singh S.P., Jain V., Chatterjee J.M. (2020). Deep Learning Solutions for Skin Cancer Detection and Diagnosis. Machine Learning with Health Care Perspective: Machine Learning and Healthcare.

[30] Paszke A., Gross S., Massa F., Lerer A., Bradbury J., Chanan G., Killeen T., Lin Z., Gimelshein N., Antiga L., Wallach H., Larochelle H., Beygelzimer A., d’Alché-Buc F., Fox E., Garnett R. (2019). PyTorch: An Imperative Style, High-Performance Deep Learning Library. Advances in Neural Information Processing Systems 32.

[31] PyTorch (2017). Finetuning Torchvision Models—PyTorch Tutorials 1.2.0 Documentation. https://pytorch.org/tutorials/beginner/finetuning_torchvision_models_tutorial.html.

[32] Wang Y., Tao X., Qi X., Shen X., Jia J. (2018). Image inpainting via generative multi-column convolutional neural networks. Proceedings of the 32nd International Conference on Neural Information Processing Systems, NIPS’18.

[33] Wang Z., Bovik A., Sheikh H., Simoncelli E. (2004). Image quality assessment: From error visibility to structural similarity. IEEE Trans. Image Process..

[34] Van der Walt S., Schönberger J.L., Nunez-Iglesias J., Boulogne F., Warner J.D., Yager N., Gouillart E., Yu T. (2014). Scikit-image: Image processing in Python. PeerJ.

[35] Achanta R., Shaji A., Smith K., Lucchi A., Fua P., Süsstrunk S. (2012). SLIC superpixels compared to state-of-the-art superpixel methods. IEEE Trans. Pattern Anal. Mach. Intell..

[36] Bradski G. (2000). The OpenCV Library. Dr. Dobb’s J. Softw. Tools.

[37] Ross A.S., Hughes M.C., Doshi-Velez F. Right for the right reasons: Training differentiable models by constraining their explanations. Proceedings of the 26th International Joint Conference on Artificial Intelligence.

[38] Du M., Liu N., Yang F., Hu X. Learning credible deep neural networks with rationale regularization. Proceedings of the 2019 IEEE International Conference on Data Mining (ICDM).

